# Overcoming Methodological Challenges for Advancing Stem Cell Therapies in Parkinson’s Disease

**DOI:** 10.1177/09636897241246355

**Published:** 2024-04-18

**Authors:** Stephen Polgar, David I. Finkelstein, Leila Karimi

**Affiliations:** 1 School of Health and Biomedical Sciences, RMIT University, Melbourne, VIC, Australia; 2 University of Melbourne, Florey Institute of Neuroscience and Mental Health, Parkville, VIC, Australia; 3 School of Medicine and Healthcare Management, Caucasus University, Tbilisi, Georgia

**Keywords:** Parkinson’s disease, stem cell therapy, methodological challenges, sham surgery, treatment algorithm

## Abstract

The quest for new and improved therapies for Parkinson’s disease (PD) remains of paramount importance, despite previous trial failures. There is a current debate regarding the potential of stem cell research as a therapeutic approach for PD. The studies of dopaminergic fetal stem cells for PD treatment, their design, and the results of the initial surgical placebo-controlled trials were reviewed in this study. Some of the fundamental methodological challenges and possible strategies to resolve them were proposed. In this article, we argue that the most important impact lies in the proof-of-principle demonstrated by clinical trials for cell replacement strategies in reconstructing the human brain. While some researchers argue that the considerable technical challenges associated with cell therapies for PD warrant the discontinuation of further development using stem cells, we believe that the opposing viewpoints are instrumental in identifying a series of methodological misunderstandings. Here, we propose to expose key challenges to ensure the advancement of the field and unlock the potential of stem cell therapies in PD treatment. Overall, this review underscores the need for further research and innovation to overcome the hurdles in realizing the potential of stem cell–based therapies for PD.

## Introduction

The prevalence of Parkinson’s disease (PD) is on the rise, leading to increased years lived with disability^1^. The global PD community emphasizes the need for research to understand the disease’s mechanisms and explore the potential benefits of new reconstructive therapies^2^. The conceptually straightforward goal of dopamine cell replacement in PD serves as an attractive starting point for the development of more complex cell replacement therapies to repair the central nervous system (CNS). Despite the absence of a current therapeutic product, clinical benefits are evident in many patients with PD transplanted with human fetal dopaminergic cells in the context of clinical trials^3^. Researchers have put forth compelling evidence-based arguments for proceeding with the project of translating dopaminergic cell transplantation into an effective treatment for PD^4^.

Developments in the field of stem cell technologies have been viewed as a game changer, offering an alternative to the logistical and ethical challenges associated with fetal cells. Stem cells hold the potential to provide a consistent and controlled supply of suitable cells for transplantation, produced in accordance with good manufacturing practices^5^. It has been proposed that the most suitable stem cells for reconstructing the nigrostriatal dopaminergic system are those which closely approximate the authentic isogenic human midbrain dopamine-releasing neurons affected by PD neuropathology^6^.

Currently, extensive international projects of preclinical studies are underway^7,8^, to uncover the mechanism of action of candidate stem cell lines and also phase 1 clinical trials to identify the optimal parameters such as patients’ age, disease duration, and symptom presentation to ensure the likelihood of significant and clinically meaningful outcomes in future trials^9^ (https://www.clinicaltrialsregister.eu/; https://clinicaltrials.gov/). While progress is being made in translating cell technology into an effective treatment of PD and a range of CNS disorders, a recent negative commentary has emerged, questioning the utility of neural reconstruction and warning against the potential risks to the lives of patients^10^. To advance stem cell–based therapies in PD, it is crucial to address the methodological challenges that hinder progress in this field. Considering the disruption to the funding and progress by the previous negative commentaries targeting fetal cell transplantation^11,12^, this article aims to critically evaluate the methodological challenges associated with the implementation and interpretation of previous research and suggest ways of resolving these challenges by identifying methodological strategies best suited to developing safe and effective reconstructive therapies for individuals with PD.

## Historical Evidence for the Efficacy of Dopaminergic Cell Transplantation

The field of dopaminergic cell transplantation has seen significant developments over the years. Initial attempts at using autologous adrenal cells for transplantation proved unsuccessful. However, subsequent phase 1, open-label clinical trials employing human fetal dopaminergic cells^13^–^15^ showed very strong levels of efficacy. For example, a study of five patients demonstrated both clinically meaningful and statistically significant improvements of 38.9% on the motor component of the Unified Parkinson’s Disease Rating Scale under no medication conditions (UPDRS motor, off), and a 61% increase in 18F-fluorodopa neural imaging from baseline, indicating both increased dopamine turnover and functional recovery following cell transplantation^13^.

It is important to note that phase 1 trials, which typically involve small patient cohorts, raise concerns about the external validity of the findings. To address this issue, meta-analyses have been conducted to integrate the multiple and diverse results, indicating strong overall weighted mean improvements in published studies, such as a 39.4% improvement from baseline on UPDRS (motor, off) under no medication conditions and an overall 40.3% increase in 18F-fluorodopa uptake^3^.

One crucial finding from the open-label studies was the significant positive correlation between improvements in motor and dopaminergic functioning. For instance, a review of key reports reported a correlation (rho) of 0.66, with *P* < 0.05, between increases in 18F-fluorodopa uptake and UPDRS (motor, off) improvements in 10 patients participating in two open-label studies^14,15^. These promising results provide a solid foundation for further advancing the project of translating cellular transplantation into effective reconstructive treatments for people with PD. It is understood, however, that the changes observed in open-label studies may not accurately represent the causal effects of the transplanted cells, given that the results of open-label trials can be influenced by confounding extraneous variables, such as placebo effects in patients or observer bias by the researchers.

Randomized controlled trials (RCTs) are considered the “gold standard” designs for establishing causal effects and evaluating the safety and efficacy of developing novel therapies^16,17^. When a developing novel treatment involves surgical procedures, most researchers argue that the implementation of confirmatory surgical placebo-controlled trials is a necessary design feature which requires sham (or imitation) surgical procedures to create the control groups. Even though relying upon sham surgery controls is ethically controversial^18^–^20^, they have been accepted as necessary to control for placebo effects and observer bias associated with evaluating the efficacy of experimental treatments of regenerative therapies requiring surgical procedures^19,21^.

In the mid-1990s, the National Institutes of Health (NIH) funded two surgical placebo-controlled trials to confirm whether the transplantation of human fetal dopaminergic cells actually caused the benefits found in open-label studies or if the benefits were influenced by placebo effects and observer bias^22,23^. Despite the therapeutic success seen in many patients participating in open-label trials, the apparent failure of two double-blind, randomized trials to report significant benefits led some researchers to believe that the transplantation approach had reached a dead end^10^. This perception, along with the costs, ethical and methodological challenges, and sustained commitment, required by researchers to complete projects for the transplantation of dopaminergic cells to achieve clear therapeutic benefits, turned the attention of many researchers’ toward exploring other promising alternate approaches to reconstructive therapies for PD^9^. It is essential to note that before the above two randomized trials were commenced, concerns were expressed that the research program of neural transplantation for PD had not progressed to a point at which implementation of randomized sham-controlled trials for confirming safety and efficacy was justified^24^. There were crucial questions regarding the optimal parameters for implementing these trials that remained unanswered in the mid-1990s. With hindsight, waiting until the results of preliminary safety efficacy trials were available might have improved the outcomes of the surgical placebo-controlled trials and generated valid decisions regarding efficacy, critical for the further development of cell transplantation for PD^13,14^.

Consistent with the issues raised above, the designs of the first two surgical placebo-controlled trials addressed research questions which are arguably more suitable for preliminary phase 1 safety and efficacy investigations^24^, rather than for designing phase 2 confirmatory trials. It would have been advantageous to have preliminary information for answering questions regarding key parameters, such as the ages of patients best suited for transplantation^22^ or the quantity of cells required for ensuring optimal benefits^23^, rather than to attempt to answer these questions within the framework of the two confirmatory surgical placebo-controlled trials, as shown in Table 1.

**Table 1.** The Design and Results of the First Two Surgical Placebo-Controlled Trials.

Freed et al.^22^	
Design	A total of 40 patients, diagnosed with PD volunteered to participate. They were assigned to two sub-groups of n=20, one with ages less than or equal to 60 years (<60) and the other over 60 (>60). Subsequently, the patients were randomly assigned into two sub-groups (n= 10), one in which they were transplanted with human fetal cells; in the other group (n=10) they underwent sham surgery.
Results	The primary outcome measure selected by Polgar and Mohamed^20^ was a subjective scale of wellbeing (−3 to 0 to +3 on a Global Rating Scale) requiring the research participants to report their subjectively estimated degree of recovery or deterioration from baseline. At the 1-year endpoint of the trial, the results were: 0.0 ± 2.1 (mean and standard deviation SD) for the transplanted group (n=20) and −0.04 ± 1.7 for the sham-operated group (n=20). In other words, neither the transplanted nor the sham-operated groups reported sufficient recovery from baseline on the Global Rating Scale and therefore the difference between the groups was not statistically significant (*P* = 0.62). Also, no benefits were evident in the sham-operated groups on any of the outcomes.
Olanow et al.^23^	
Design	34 patients with PD volunteered to participate and were randomly assigned to three groups, one group transplanted with mesencephalic cells from 4 donors (*four-donor group*), another group with 1 fetal transplant per (*one-donor group*) side, and the third group underwent sham surgery.
Results	The primary outcome measure selected by Kim et al.^21^ was assessed as the change from baseline to the 2-year endpoint of the trial, the UPDRS (motor, off) with the positive values indicating deterioration and negative values improvement in motor symptoms. The results were mean (and standard deviation) change at trial endpoint (2 years) from baseline: 4.1(± 5.5) for the one donor group (n=11), −0.4(±2.8) for the four-donor group (n=12), 8.4(±5.5) for the sham-operated control group (n=11). Analysis of covariance did not show significant differences across the three groups (*P* = 0.244). It should be noted that the sham-operated control group demonstrated significant deterioration from baseline at the 2-year endpoint, (*P* < 0.05) indicating the complete absence of a placebo response, also no evidence for a placebo effect based on the 18F-fluorodopa imaging.

Although the two surgical placebo-controlled trials failed to demonstrate at the designated trial end points statistically or clinically significant benefits on the primary outcomes selected by the researchers for the transplantation of dopaminergic fetal cells, their results do not necessarily constitute valid evidence for the failure of the entire research program. It was recognized by the researchers that it was a mistake to rely on the subjective “Global Rating Scale” as the primary outcome measure for evaluating treatment efficacy^25^. When data were analyzed on UPDRS (motor, off), statistically significant differences (*P* = 0.0003) were found for the transplanted group with patients 60 years or younger as compared with the sham-operated controls^25^. Also a significant effect size was evident in the second trial^23^, within a group of patients receiving four fetal tissue transplants per side up to 6–9 months following transplantation, although this benefit gradually faded to no effect from baseline at the 2-year endpoint of the trial, possibly due to the premature cessation of immunotherapy.

The implication which may be drawn from the above two confirmatory trials when their methodological limitations are taken into account is that although the transplantation of fetal cells may not work for all subgroups of patients with PD under all circumstances, the overall results from phase 1 and phase 2 studies clearly provide “proof of concept” for the potential benefit of cell transplantation. It is unfortunate that following the mistaken inference of “futility,” no new RCTs have been published for the last two decades, resulting in a dearth of new trial results for making valid decisions regarding the safety and efficacy of the implantation of dopaminergic fetal cells^26^.

## Evidence for the Magnitude of Placebo Effects and Responses in Surgical Placebo-Controlled Trials

The perceived failure of cellular therapies under surgical placebo-controlled trials designs has been attributed to the powerful influences of observer bias and placebo effects, inflating the apparent benefits of treatment outcomes^17,27^. It has been suggested that given the perceived demands associated with participating in an experimental neurosurgical procedure and the strong expectations of treatment benefits, it follows that, “. . . consenting to undergo experimental brain surgery elicits the largest and most durable placebo response and therefore must be controlled for with sham procedures”^10^.

Distinguishing between the concepts of placebo response and placebo effect is important; the response refers to the measured changes on specific outcome measures in the control group, while the effect represents inferences drawn regarding the true therapeutic benefits of the sham procedures^28^. It is important to note that no evidence emerged in the two ground-breaking surgical placebo-controlled trials for improvements on either the primary or the secondary outcome measures for clinical improvements in the sham-controlled groups^22,23^ (see Table 1). Further, a later meta-analysis reported that a combined weighted average placebo responses of the sham-controlled groups across nine published surgical placebo-controlled trials were found to be 4.3 units, with 95% confidence interval (CI) of 3.1–5.6 on UPDRS (motor, off)^29^. Because this magnitude of improvement in PD patients has been established as a modest to moderate response^30^, we suggest that it probably would not exert a decisive influence on the large, clinically significant improvements from baseline in successfully transplanted groups postulated as approximately 30%–50% or >20 points on UPDRS (motor, off)^29^.

There is convincing evidence that placebo effects are causally associated with not only increased activity of dopaminergic neurons regulating motor functioning but are also involved in mediating the research participants’ expectations of reward^31,32^. However, the evidence for the association between improved motor functioning and neural imaging indicating increased dopamine activity in placebo-controlled groups has been obtained in the context of pharmacological trials^31^. The aims and the timelines at the endpoints of the symptomatic pharmacological treatments are quite different from those of confirmatory trials of surgical cell transplantation. Consistent with the earlier findings, the results of a recent meta-analysis of sham surgical procedures have not shown statistically significant and clinically meaningful benefits based on positron emission tomography (PET) scan assessments indicating the absence of structural reconstruction^29^.

RCTs are implemented to subtract the results of the control sham-operated group from active group to identify the true unbiased value of the efficacy of the intervention. A relevant question is why is it that in sham surgery-controlled trials, there are variable outcomes, some of which are successful in showing therapeutic benefit and others not?^11^. The reduced overall performance of some of the patients under sham surgery-controlled double-blind designs^31^ may be attributed to the diminished potential contribution of patients in the transplanted groups to the effective integration of the dopaminergic grafts compared with open-label trials^33^.

A question which emerges from the above analysis is why the transplanted fetal cells generated marked functional benefits in some patients, while in other cases, the cells survived but there were no clinical improvements. To address this question, more evidence is needed from well-defined stem cell lines (good manufacturing practice) in well-characterized subpopulation of people living with PD. Recently, a number of new biomarkers for PD have been established, for example, an olfactory test combined with CSF-derived alpha-synuclein seed amplification assay that may help select a more well-defined group of patients with PD^34^.

## Lessons From the Design and Implementation of Previous Clinical Trials

Currently, it is widely accepted that the neuropathology of PD extends beyond the loss of nigrostriatal projections^35^ and encompasses multisystemic synucleinopathy of the human nervous system with a range of functional consequences that extend beyond the nigrostriatal system^36^. It follows that the reconstruction of the nigrostriatal system alone will not provide a cure, but if it is successful, will be of lasting clinical and life-changing benefit to people experiencing the disabling symptoms of PD.

In the past three decades, deep brain stimulation (DBS) has emerged as an effective and clinically adopted therapeutic strategy for controlling motor symptoms in PD^37^. Because DBS has emerged as an effective, evidence-based neurosurgical treatment for PD, it would serve as a more relevant comparator group than sham-operated controls when designing future trials for evaluating the real-life benefits of stem cell transplantation. A well-functioning health care system should offer a range of effective treatments that may not cure targeted chronic diseases but rather ensure the best possible quality of life for people living with disabling, progressive CNS disorders, such as PD^38^.

Most importantly, the development of a combination therapies acting in synergistic manner may ultimately yield the most favorable therapeutic outcomes^39^. For example, currently, the majority of patients undergoing successful DBS treatment may also receive drug treatment and rehabilitation as needed. There are many possible combinations, such as providing posttransplantation-targeted rehabilitation programs to facilitate the integration of grafted cells within the brain^40,41^. The challenge is to leverage the potential of stem cells to enhance the benefits previously observed in the transplantation of human fetal dopaminergic cells.

Rather than seeking a specific best practice treatment or a panacea, the alternative view is to develop of a range of available treatments for clinicians to address the needs of individual patients as determined by factors like age at diagnosis, severity or duration of the condition, cognitive and autonomic dysfunction, and financial resources of the patients and their communities. The optimal outcomes for the PD patient population can be best achieved by the development of treatment algorithms, similar to those used for pharmacological management of PD^42,43^. A realistic aim for stem cell–based research is to provide novel treatment approaches for subgroups of patients who are most likely to benefit from the long-term reversal of dopaminergic cell loss, improvement of motor symptoms, and ultimately, to provide clear, both objective and subjective evidence for enhanced quality of life^44^.

## Summary and Conclusion

**Figure 1.**
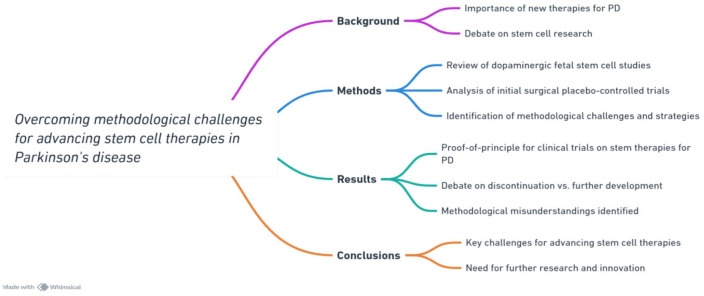
A summary fo the review.

In summary, the current state of research on reconstructive therapies using cell transplantation for PD indicates that the degree and duration of benefits vary significantly among individual patients. The mixed results for an emerging complex therapy do not indicate that we should discontinue further exploration of cell transplantation; rather, they emphasize the need for more work to be done to improve conditions that promote stable functional connections of transplanted cells^45,46^. Figure 1, provides a summary of the review.

The recent trial conducted by TRANSEURO (2019) illustrates the multiple challenges—ethical, logistical, financial, and professional dedication—confronting researchers undertaking clinical trials to evaluate the safety and efficacy of cell transplantation for PD^47^. Insufficient data and inadequate statistical power to analyze the trial results have contributed to mistaken conjectures such as calling for an end to stem cells research on the grounds that the previous trials with fetal mesencephalic cells were “futile”^10,11^.

While there is unequivocal evidence of placebo effects and responses in clinical trials evaluating the efficacy of pharmacological products for PD, the evidence emerging from the sham surgical arms of trials assessing the efficacy of cell transplantation remains disputed. A recent systematic review and meta-analysis of published phase 2 trials^29^ found no convincing evidence for placebo effects and reported overall modest to moderate placebo responses, challenging the methodological necessity of imposing sham surgical procedures on patients with PD volunteering as trial participants.

The discussion in this review highlights the great challenges associated with the reconstruction of the human brain and suggests methodological approaches for translating the ground-breaking stem cell research discoveries into effective PD treatments. Table 2 presents some of the fundamental methodological challenges and possible strategies discussed in this article to resolve them. Overall, this review underscores the need for further research and innovation to overcome the hurdles in realizing the potential of stem cell–based therapies for PD.

**Table 2.** Addressing the Methodological Challenges.

Methodological challenges	Possible resolution
It is difficult to independently analyze trial results or mine combined data sets from completed trials.	Construction of de-identified public databases and repositories should be mandatory and accessible to researchers in the field
Current data strongly suggests that sham surgical placebo controls are unnecessary as placebo effect size has been established. The inclusion of a surgical placebo diminishes the power of the study by reducing the size of the treatment arm.	Future evaluative pragmatic trials should rely on comparison with established published natural history cohorts and effectiveness of therapies such as deep brain stimulation or dopamine replacement therapies.
Recent understandings from cell replacement trials indicate that they are not likely to be a cure. What is not understood is which people from this heterogenous syndrome would benefit from this therapy.	Previous trials have shown long term benefits of cell transplants. Much work is currently going on to see which patients would benefit from the enhanced cell therapies, what synergistic treatments could be integrated, and which are the optimum cells for transplant. Development of a treatment algorithm would assist patients, surgeons, clinicians, and neurologists in this decision.
